# Disentangling metabolic and neurovascular timescales supporting cognitive processes

**DOI:** 10.1073/pnas.2506513122

**Published:** 2025-09-22

**Authors:** Francesca Saviola, Stefano Tambalo, Laura Beghini, Asia Ferrari, Barbara Cassone, Dimitri Van De Ville, Jorge Jovicich

**Affiliations:** ^a^CIMeC, Center for Mind/Brain Sciences, University of Trento, Rovereto 38068, Italy; ^b^Department of Medical and Surgical Specialties, Radiological Sciences and Public Health, University of Brescia, Brescia 15123, Italy; ^c^Neuro-X Institute, Ecole Polytechnique Fédérale de Lausanne, Geneva 1202, Switzerland; ^d^Department of Physics, University of Torino, Torino 10125, Italy; ^e^Department of Molecular Biotechnology and Health Sciences, University of Torino, Torino 10124, Italy; ^f^Department of Physics, Faculty of Natural Sciences, Norwegian University of Science and Technology, Trondheim 7034, Norway; ^g^Department of Clinical and Experimental Sciences, Neurology Unit, University of Brescia, Brescia 15123, Italy; ^h^Department of Psychology, University of Milano-Bicocca, Milan 20126, Italy; ^i^Department of Radiology and Medical Informatics, University of Geneva, Geneva 1202, Switzerland

**Keywords:** functional magnetic resonance spectroscopy, time-varying functional connectivity, excitation–inhibition balance

## Abstract

The balance between neural excitation and inhibition (EIB), governed by glutamatergic and GABAergic neurotransmission, is an essential mechanism supporting cognitive processes. Yet, little is understood about how EIB shifts with engaging cognitive load and its impact on functional connectivity dynamics. Here, we combined time-resolved functional magnetic resonance spectroscopy and magnetic resonance imaging to investigate temporal profiles of the reciprocal modulation between EIB and functional network dynamics during working memory tasks in healthy subjects. We quantified EIB kinetics by measuring excitatory (Glx) and inhibitory (GABA+) levels and assessed their temporal coupling with dynamic functional connectivity states, revealing a dependence that scales with cognitive load. Notably, we found that as cognitive challenges intensify, brain networks transition toward more specialized and enduring connectivity configurations, accompanied by imbalances that favor excitation, changes that may impact cognitive adaptability. Fluctuations in EIB and functional connectivity occurred on comparable timescales and were both associated with individual differences in cognitive performance. Importantly, this experimental approach demonstrates a close synergy between EIB kinetics, brain network dynamics, and cognitive performance, defining the groundwork for exploring healthy and aberrant cognitive states.

## Significance

This study reveals how excitation/inhibition balance (EIB) kinetics and functional brain network dynamics coevolve during cognitive challenges. Using time-resolved spectroscopy, we demonstrate that neural EIB fluctuations and functional connectivity operate on comparable timescales. Sustained cognitive load drives EIB imbalance and temporally invariant network states. Notably, neurochemical and vascular dynamics exhibit synchronized oscillations, suggesting a shared regulatory mechanism for maintaining cognitive flexibility. These findings bridge molecular, systems, and cognitive neuroscience by linking cellular EIB mechanisms to network-level cognitive processes, providing insights into the neural basis of cognitive adaptability.

The functionality of the human brain hinges on a delicate balance between two principal neurotransmitters: glutamate, the primary excitatory neurotransmitter, and γ-aminobutyric acid (GABA), with an inhibitory effect on neuronal excitability (1). Their ratio, known as excitation–inhibition balance (EIB), is crucial for maintaining cognitive functions and synaptic plasticity (2–4). Disruptions in EIB are implicated in numerous neurological and psychiatric disorders, highlighting its critical role in both brain health and disease (5). While substantial research has focused on the static roles of excitatory and inhibitory circuits, there is growing recognition of their dynamic interplay and its significance in human cognition (6). However, fundamental questions remain unresolved: How do EIB dynamics affect the functional connectivity of networks supporting cognitive processes? And critically, given ongoing debate about the timescales of these dynamics, do changes occur at rapid (seconds) or prolonged (minutes) intervals?

Preclinical studies have demonstrated both the feasibility and the cognitive relevance of in vivo EIB mapping (7–9). However, noninvasive EIB estimation in humans remains challenging, pushing the field to advance through the development of computational approximation models (10). Indeed, these proxies probed how microscale human brain organization strongly relies upon hierarchical computationally derived EIB gradients (11–13) with a strong association to blood oxygenation level-dependent (BOLD) signal temporal variability (14), both in health and disease (5, 15, 16). All of the above stated the significance of the relationship between time-varying patterns of functional connections and EIB kinetics, which urge to be deepened through more reliable estimations of the latter.

A growing array of methods, ranging from biophysical modeling to signal-derived statistical metrics, have been used to estimate EIB in the human brain. Among these, Magnetic Resonance Spectroscopy (MRS), particularly in its edited and functional variants, offers a unique, noninvasive means to quantify region-specific concentrations of glutamate and GABA+, providing neurochemical proxies of EIB that complement, rather than replace, computational and functional surrogate approaches (e.g., EEG, MEG, fMRI-derived). Within this context, recent modeling approaches have revealed complex temporal dynamics of neurometabolites as measured by functional MRS (fMRS). Two contrasting hypotheses emerged, each proposing distinct timescales for metabolite fluctuations. Lea-Carnall et al. model (17) suggested rapid shifts in glutamate and GABA levels between vesicular and cytosolic compartments, occurring within seconds of neural activity changes. In contrast, Mangia et al. hypothesis (18) argued for slower processes linked to energy metabolism and operating over several minutes. These divergent perspectives highlighted the multifaceted nature of fMRS signals, which likely encompass a spectrum of temporal dynamics from rapid neurotransmitter cycling to gradual metabolic adjustments. The relative contributions of these fast and slow processes may vary depending on brain regions and experimental conditions, underscoring fMRS data complexity.

This dynamic complexity is particularly relevant to understanding brain functions like working memory (WM), a core component of higher-order cognition and goal-directed behavior. WM significantly rests on prefrontal cortices, where glutamatergic pyramidal cell firing underpins the maintenance of information (19) and GABAergic circuit disruptions can result in severe deficits (20). Recent studies have begun to reveal that the neural coding underlying WM is itself dynamic, with coding formats and timescales adapting flexibly to varying cognitive loads and exhibiting laminar-specific modulation across the trial (21). In parallel, Wolman et al. (22, 23) provide compelling evidence that neural timescales may serve as a mechanistic bridge linking input dynamics, intrinsic neural processing, and cognitive output (i.e., WM capacity). Distinct cortical territories, from primary to prefrontal regions, exhibit characteristic timescale properties directly associated with individual differences in WM performance. This underscores the importance of both input-driven dynamics and the flexibility of neural timescales in supporting WM. Such perspectives resonate with the broader frameworks (24) conceptualizing how large-scale networks as inherently flexible and adaptive architectures underlying intelligent behavior. Despite these theoretical advances, there is currently no empirical evidence of human dynamic regulation of EIB during WM tasks, and it remains unclear how changes in neurometabolic substrates relate to time-varying functional connectivity in prefrontal cortices.

To address this gap, we introduced an innovative experimental approach designed to investigate a key cognitive neuroscience question—WM modulation under varying cognitive loads—through the integration of functional neuroimaging (fMRI) and molecular mechanisms (fMRS). The advancement of the protocol is two-fold. First, it involved interleaving functional MRI with functional MRS (25–28), enabling noninvasive examination of brain connectivity and neurometabolite coupling as a function of cognitive load. Second, we adapted fMRS to estimate EIB kinetics for increasing cognitive load. We speculate that this protocol opens the path to applications in various domains, such as basic cognitive or clinical neuroscience research as well as validation and refinement of computational models that provide mechanistic links between neurobiological activity and behavior in health and disease.

To demonstrate the feasibility of our protocol, we applied it to healthy young volunteers using a clinical 3 T MRI system. Specifically, we explored the hypothesis (29) that different mental workloads during WM tasks elicit parametric alterations of EIB kinetics, reflecting fluctuations in executive functional networks over time. We envision that metabolic and vascular changes occur on differential timescale under the same WM tasks, with slower changes detected by BOLD signaling compared to the faster rates of neurochemical responses, elicited primarily by local excitatory glutamatergic transmission that reflects cognitive demand. Specifically, our first aim is to detect alterations in neurometabolites, especially excitatory and inhibitory ones, across sessions characterized by different levels of cognitive load. The second aim involves moving beyond static estimation of neurometabolite concentrations to investigate their cycling during the fMRS time course, offering insights into the timescale of responses. Last, our third objective is to compare and integrate the time-resolved contributions of metabolic and vascular information in sustaining goal-directed behavior. To accomplish this, we employed time-varying models of functional connectivity and assessed associations with metabolic data.

## Results

### In Vivo Detection of EIB Kinetics.

Out of 25 subjects with fMRS acquisitions, only 12 met the stringent data quality criteria. Indices of spectral quality and the group-averaged edited GABA+ and Glx spectra from the left DLPFC (gray matter fraction: 0.52±0.04%) are detailed in the *SI Appendix*, *Supplementary Materials and Methods*. Notably, static fMRS data analysis showed no significant WM load effects on normalized concentration measures averaged during each cognitive condition for all metabolites.

**Fig. 1.**
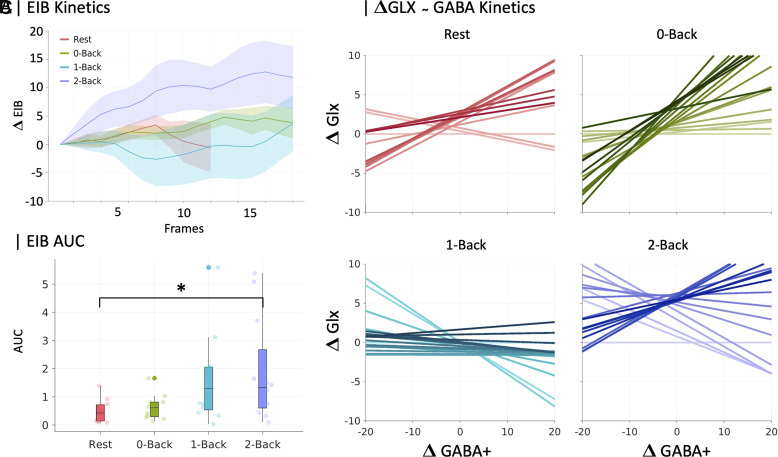
Changes of in vivo EIB kinetics across cognitive load levels. (*A*) Kinetics of EIB curves using a sliding-window approach (4 min per frame, with 12-seconds shifts) for each cognitive condition. Thick lines show the mean, and the shaded areas the SEM. (*B*) AUC Boxplot derived from A depicting Load effects between Rest and 2-Back sessions. (*C*) Evolution of Glx/GABA+ imbalance to reveal the directionality of the effect. Temporal dimension is depicted as a color gradient from light (first frame) to solid (last frame).

In contrast, dynamic analysis revealed more nuanced results (Fig. 1*A*): EIB area under the curve (AUC) indicated a significant effect of Load [χ^2^(3,44) = 9.95, *P* = 0.02, Fig. 1*B*]. Post hoc comparisons showed that the 2-Back condition had a significantly higher EIB curve increase compared to the resting-state condition [Rest > 2-Back, *P* = 0.04, Mean Ranks Difference = −14.9, Fig. 1*B*]. A significant effect was also observed for GABA+ [χ^2^(3,44) = 10.64, *P* = 0.01], while no significant effects were found for Glx across different frames.

The observed increase in EIB during the high cognitive load condition (2-Back) suggests a potential adaptive mechanism in brain metabolism to meet increased cognitive demands. These findings underscore the importance of monitoring the evolution of neurometabolite concentration, as they reveal task-related modulations not evident in static measures.

### Comparing Executive Network Temporal Dynamics and EIB Kinetics.

**Fig. 2.**
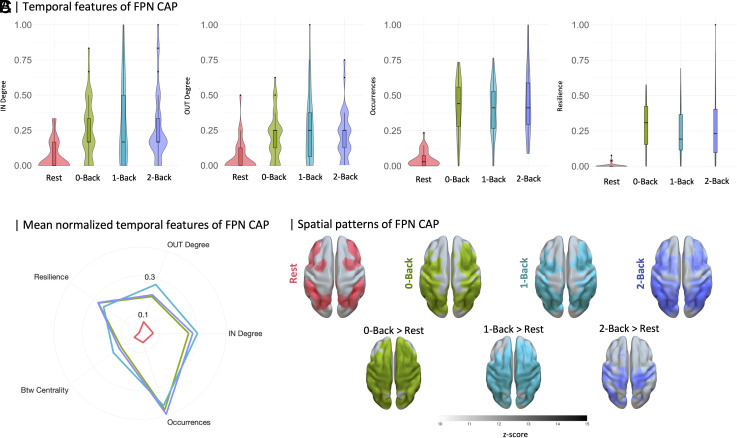
Executive network temporal dynamics moving toward stability as a function of cognitive load. (*A*) violin plots of normalized FPN-CAP temporal properties exhibiting significant changes across sessions (*P* < 0.001). (*B*) radar plot displays the level of temporal dynamic properties of frontoparietal networking over the time series. Temporal properties are represented as normalized scores ranging 0 to 1 with respect to data distribution across runs. (*C*) Voxel-wise differences of backprojected FPN-CAP. The first row depicts one-sample *t* test results for the main effect of sessions. The second row shows pairwise *t* test across sessions (FWE across voxels and Bonferroni across comparisons *P* < 0.016).

The FPN seed-based dynamic fMRI analysis identified four FPN-CAPs across all sessions, consistently observed in both the full sample of 36 subjects and a subset of 12 subjects with concurrent MRS acquisition. These FPN-CAPs, matched to the Yeo atlas (30) using cross-correlation (r-value > 0.3), exhibited distinct temporal patterns corresponding to varying cognitive loads (*SI Appendix*, *Supplementary Materials and Methods*). Table 1 summarizes the temporal properties of FPN-CAP networking for the full sample (MRS subset in *SI Appendix*, Table S4). Both tables reveal significant differences in higher cognitive load conditions compared to resting-state (Fig. 2). Notably, FPN-CAP temporal dynamics increased significantly as mental workload rose, stabilizing during higher cognitive task conditions.

**Table 1.** Temporal properties derived from CAPs analysis of FPN among different sessions in fMRI sample (N = 36). The table reports key temporal metrics, including occurrences and other graph-derived metrics for each session. Differences across condition show the results of repeated-measures statistical analysis, indicating whether the temporal properties significantly differed over time

Temporal FPN properties	Working memory conditions				Differences across conditions (FDR corrected)		
	rest	0-back	1-back	2-back	Post hoc comparisons	*P*-value	Chi-square/ A-B estimate
Occurrences (%)	1.7 ± 1.9	14.3 ± 6.4	13.5 ± 6.2	15.1 ± 7.7	0-back > rest 1-back > rest 2-back > rest	<0.001** <0.001** <0.001** <0.001**	69.4 66.9 62.5 69.1
Resilience	0.0007 ± 0.002	0.04 ± 0.02	0.03 ± 0.03	0.04 ± 0.03	0-back > rest 1-back > rest 2-back > rest	<0.001** <0.001** <0.001** <0.001**	65.75 65.75 61.06 64.43
Betweenness centrality	0.2 ± 0.4	0.6 ± 0.9	0.9 ± 1.3	0.7 ± 1.2		>0.05	
IN Degree	0.002 ± 0.003	0.008 ± 0.006	0.009 ± 0.009	0.008 ± 0.008	0-back > rest 1-back > rest 2-back > rest	<0.001** <0.001** <0.001** <0.001**	25.35 37.83 39.00 38.94
OUT Degree	0.003 ± 0.004	0.008 ± 0.007	0.010 ± 0.010	0.009 ± 0.007	0-back > rest 1-back > rest 2-back > rest	<0.001** <0.001** <0.001** <0.001**	25.93 35.98 42.87 37.83

Spatial topographies of FPN-CAP also showed significant voxel-wise changes across sessions (*P*-value_FWE_ < 0.016, Bonferroni corrected). The activation pattern evolved from anatomically confined areas during resting-state to a more extensive distribution under higher cognitive loads. In the 2-Back condition, this expanded pattern encompassed all key regions of the FPN, with notably increased involvement of lateral prefrontal areas. Pairwise comparisons further confirmed these findings, revealing larger differences between resting-state activations and those during attentive (0-Back) or lower load (1-Back) conditions. This progression highlights the FPN’s adaptive engagement in response to increasing cognitive demands.

To integrate neurovascular information from dynamic functional connectivity with Glx/GABA concentration kinetics, we analyzed visibility graph-based time series properties of the EIB curve. Specifically, we focused on out-degree (reflecting a temporal gradient of AUC) and Kullback–Leibler divergence (KLD; an index of temporal reversibility) across all conditions (*SI Appendix*, Table S5). A Kruskal–Wallis nonparametric test across sessions revealed statistically significant differences in the out-degree of EIB [χ^2^(3,44) = 9.92, *P* = 0.02]. Post hoc comparisons indicated an increased out-degree of EIB in the 1-Back condition compared to resting-state (Rest < 1-Back, *P* = 0.02, Mean Ranks Difference = −16.5), suggesting a change in the temporal dynamics of EIB with increasing cognitive load.

While no significant differences were found in KLD, an increasing trend was observed as a function of cognitive load. The comparison between Rest and 2-Back conditions showed a qualitative, nonsignificant trend (Rest<2-Back, Mean Ranks Difference = −12.37, *P* = 0.06) toward temporal irreversibility in EIB under higher cognitive demands.

These findings highlight the complex relationship between cognitive load and EIB kinetics: The increased out-degree in the 1-Back condition suggests more frequent changes in EIB levels, while the rising KLD trend under higher cognitive loads indicates time-dependent changes in EIB. Together, these results imply that EIB-related metabolic processes and functional network reorganization are adaptive and reciprocal, responding dynamically to varying cognitive demands.

### Behavioral Performance Results.

Behavioral measures (i.e., RTs, accuracy, and *d’*) indicated a significant effect of load across sessions (*P* < 0.05; see *SI Appendix*, *Supplementary Materials and Methods*) for all variables of interest. This supports the notion that cognitive load increases across sessions (31), suggesting corresponding changes in brain functional connections and neurometabolism.

### The Effect of EIB Kinetics and Executive Networking on Behavioral Performance.

Our analysis revealed complex relationships between neurometabolites, FPN temporal properties, and task performance. The Generalized Linear Model (GLM), highlighted a significant role of static GABA+ concentration in predicting reaction times (RTs) (β = −0.1, *P*-value_FDR_ < 0.001; see *SI Appendix*, *Supplementary Materials and Methods*). However, neurometabolite kinetics showed no significant effect on behavioral performance. While previous research has linked elevated GABA levels to improved task performance measured by accuracy (32, 33), our findings did not demonstrate a significant relationship between accuracy and GABA+. Instead, the significant association with RTs suggests that higher GABA+ concentrations may enhance readiness and attentional capacity during task execution, resulting in faster RTs. When examining the relationship between trial-by-trial behavioral performance and neurometabolite dynamics using linear mixed-effects models, we found that both EIB and GABA AUCs exhibited significant negative associations with performance specifically in the 2-Back condition (EIB: β = −0.3, R^2^ = 0.8, *P* < 0.001; GABA: β = −0.6, R^2^ = 0.6, *P* < 0.01), while no significant relationships were observed for Glx or in lower-load conditions, indicating that these EIB-related metabolite markers become most predictive of performance as WM demands increase. Expanding on these findings, models incorporating metabolite-by-task interactions revealed significant positive associations between dynamic Glx concentration and performance in both the 1-Back (β = 0.8, R^2^ = 0.1, f^2^ = 0.03, *P* < 0.01) and 2-Back (β = 0.6, R^2^ = 0.1, f^2^ = 0.03, *P* < 0.01) conditions. This pattern suggests that elevated Glx levels may support cognitive resilience as WM demands increase. Furthermore, a significant positive interaction was observed for GABA in the 2-Back condition (β = 0.5, R^2^ = 0.1, f^2^ = 0.01, *P* < 0.05), indicating that dynamic increases in GABA can also be associated with improved performance under maximal cognitive challenge. Notably, no significant interactions were detected for EIB in these dynamic models, highlighting a divergence between static and dynamic neurochemical predictors. Importantly, while static GABA+ concentrations were associated with faster reaction times, only the dynamic (AUC-based) measures of EIB and GABA revealed strong negative associations with behavioral performance under high cognitive load. Conversely, the dynamic modeling approach also uncovered beneficial effects of Glx and GABA fluctuations on performance that were not apparent in static analyses. This contrast underscores the importance of capturing trial-by-trial neurochemical fluctuations, as these dynamic measures become particularly relevant for predicting individual differences in WM performance when task demands are elevated.

Furthermore, FPN-CAP temporal properties exhibited significant associations with cognitive performance. Three key dynamic features emerged as predictors: in-degree (likelihood of entering an FPN state), out-degree (likelihood of exiting an FPN state), and occurrences (frequency of a given state). For *d’*^’^, significant effects were observed for out-degree as a fixed effect (β = 7.9, *P*-value_FDR_ < 0.001) and in interaction with 1-Back (β = −18.6, *P*-value_FDR_ < 0.001) and 2-Back sessions (β = −5.2, *P*-value_FDR_ < 0.001). Accuracy was significantly affected by out-degree interacting with the 1-Back session (β = −13.9, *P*-value_FDR_ < 0.001), in-degree interacting with both 1-Back (β = −9.8, *P*-value FDR < 0.05) and 2-Back sessions (β = −11.5, *P*-value_FDR_ < 0.05), and occurrences interacting with both 1-Back (β = −0.005, *P*-value FDR < 0.05) and 2-Back sessions (β = −0.005, *P*-value FDR < 0.05; see *SI Appendix*, *Supplementary Materials and Methods*). Notably, no significant effects were found for RTs. The negative relationship between behavioral performance and FPN-CAP temporal properties suggests that during the 1-Back and 2-Back runs, task performance may have declined as FPN-CAP properties increased. This instability within the FPN-CAP may indicate that heightened engagement of the network could lead to variations in cognitive control and attentional resources, contributing to challenges in maintaining optimal task performance.

Examining the relationship between FPN-CAP temporal properties and static neurometabolite concentrations revealed significant effects exclusively for GABA+. All assessed FPN-CAP temporal properties significantly predicted GABA+ levels, particularly in interaction with the 0-Back session. Significant interactions were observed for out-degree (β = −0.2, *P*-value_FDR_ < 0.01), in-degree (β = −0.2, *P*-value_FDR_ < 0.01), occurrences (β = −0.2, *P*-value_FDR_ < 0.01), betweenness centrality (Btw) (β = −0.2, *P*-value_FDR_ < 0.01), and resilience (β = −0.2, *P*-value_FDR_ < 0.01; see *SI Appendix*, *Supplementary Materials and Methods*).

### The Cognitive Modulation of Temporal Dynamics and Its Top–Down Effects on EIB Kinetics.

To deepen our understanding of the relationship between Glx/GABA kinetics and FPN-CAP temporal features across WM cognitive loads, we conducted a Partial Least Squares Correlation (PLSC) analysis. This analysis focused on a subset of subjects with MRS measurements (N = 12), utilizing visibility graph properties of EIB curves. We performed the CAP analysis by concatenating data from all 12 subjects across different sessions, using the left DLPFC as a consistent seed region (*SI Appendix*, *Supplementary Materials and Methods*). PLSC was selected for its ability to integrate multimodal brain properties, identifying optimal linear combinations of CAP temporal features and EIB kinetics features that maximally correlate.

**Fig. 3.**
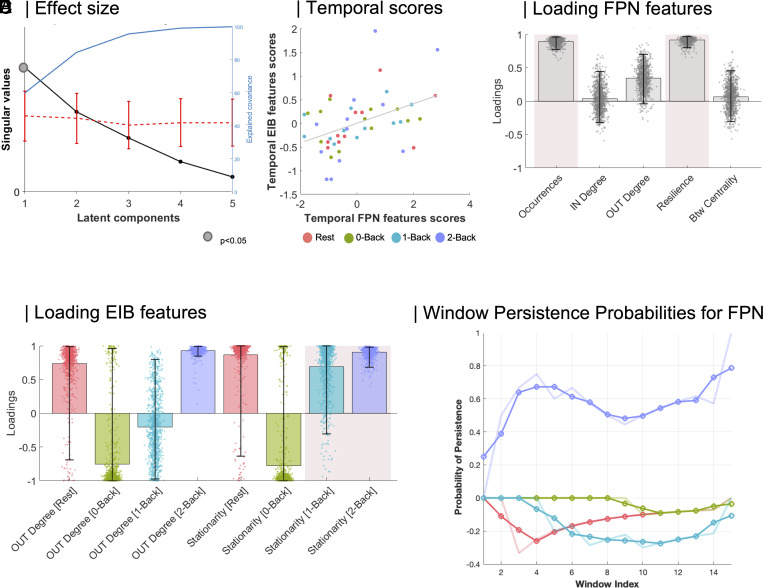
Dynamics of neurometabolite concentration contribute to constrain temporal reconfiguration of cognitive states. (*A*) Partial least squares analysis (PLS) was used to assess the multivariate relationship between EIB kinetics temporal features and executive network (FPN) temporal features. PLS identified a single significant latent variable (Covariance explained = 59%, dotted *P*-value = 0.03; two-tailed). The line plot describes the median, and bounds represent the first (25%) and third (75%) quartile of the distribution. (*B*) Temporal patterns of EIB and FPN scores are depicted for the first Latent variable. The two brain score patterns are significantly correlated (*P* = 0.0022, r = 0.43; two-tailed). R-values denote the Pearson correlation coefficient, and the linear regression line collapsed across groups is added to the scatterplot for visualization purposes only. (*C*) PLS loading FPN features are related to all temporal properties of executive networking and are depicted as bar charts with their corresponding 95% CI from 10,000 bootstrap resampling. Pink boxes describing reliable loading are plotted for significant bootstrap testing. Spatial distributions of executive CAP networking are depicted across the cortex for each cognitive workload condition. (*D*) PLS loading EIB features are shown for each workload condition as bar charts (a single PLS loading per EIB temporal scores for each session) with their corresponding 95% CI from 10,000 bootstrap resampling. Pink boxes describing reliable loading are plotted for significant bootstrap testing. (*E*) fMRS-matched sliding-window approach to investigate time scale similarities of FPN-CAP with EIB kinetics. Abbreviations: frontoparietal network (FPN), coactivation pattern (CAP), betweenness centrality (Btw), excitation–inhibition balance (EIB).

The PLSC analysis yielded a significant Latent Component (LC) as determined by permutation testing (covariance explained = 59%, *P* = 0.03). This LC captured the greatest covariance between FPN temporality and EIB kinetics. Fig. 3*B* illustrates the maximization of correlation between brain scores achieved by the analysis (r = 0.43, *P* = 0.002). We then examined the corresponding FPN-CAP and EIB kinetics temporal feature loadings across sessions, identifying the features that contributed most significantly to the spatial patterns captured by the first LC (Fig. 3 *C* and *D*). This integrated analysis provides a comprehensive view of how FPN dynamics and EIB kinetics covary across different cognitive loads, offering insights into the neural mechanisms underlying WM processes. The significant LC and strong correlation between brain scores emphasize the close coupling between network dynamics and neurometabolite fluctuations during cognitive tasks.

Our analysis revealed diverse correlations between temporal properties of EIB kinetics and FPN dynamics across sessions, particularly emphasizing occurrences and resilience, suggesting their potential role in sustaining neurotransmission. The temporal gradient of the EIB curve (represented by average EIB out-degree) and its stationarity (interpreted as the inverse of KLD) exhibited characteristic associations with FPN temporal properties. As cognitive load increased parametrically, both EIB out-degree and stationarity initially showed negative associations with temporal FPN properties, primarily driven by occurrences and resilience (28). However, this relationship shifted toward a positive association under higher mental workload conditions.

In summary, our analysis indicated that brain weights were particularly robust for EIB kinetics in terms of stationarity across sessions. This finding suggests that the correlation weights were heavily influenced by the KLD of EIB. As subjects progressed through different cognitive states, the EIB curve became less time-invariant (i.e., less stable) under higher workload conditions. As cognitive demands increased, the brain appeared to transition from a more stable EIB state to a more flexible one, potentially allowing for greater adaptability in neural processing.

To gain deeper insights into the temporal scale similarities between the two measurements, we analyzed the probability of persistence for the given FPN-CAP across EIB-matched time intervals (i.e., window indices; Fig. 3*E*; see Supplementary Material for other CAPs). A Kruskal–Wallis test on persistence curve points demonstrated a significant effect of Load [χ^2^(3,56) = 38.9, *P* < 0.001; Fig. 3*E*]. Post hoc comparisons yielded particularly interesting results for the 2-Back condition, which showed a significantly higher persistence probability curve compared to other conditions: i) Rest > 2-Back (*P* < 0.001, Mean Ranks Difference = −32.2); 0-Back > 2-Back (*P* < 0.01, Mean Ranks Difference = −20.2); 1-Back > 2-Back (*P* < 0.001, Mean Ranks Difference = −35.2). These findings suggest that the 2-Back condition, representing the highest cognitive load in our study, is associated with a distinct pattern of FPN-CAP persistence. The increased persistence probability in this condition may indicate more stable or sustained engagement of relevant brain networks during high-demand cognitive tasks. This observation provides valuable insights into how brain dynamics adapt to meet increasing cognitive challenges, potentially reflecting a shift toward more focused and sustained neural activity patterns under higher cognitive loads.

## Discussion

The temporal interplay between excitatory and inhibitory compartments supporting high-order cognitive functions remains a pivotal point for a comprehensive understanding of the human brain. In this respect, the time-dependent mapping of EIB enabled by fMRS represents a unique window on the in vivo dynamics of neurochemical signaling. By introducing a cutting-edge experimental design to integrate functional neuroimaging and spectroscopy, we provide compelling evidence that a temporal link between the two exists, grounded on regulatory principles. This relationship involves two key points: First, the differential temporal cascades between EIB-related metabolic compartments are associated with a dynamic reconfiguration of cortical connectivity features, such as stable dynamics; second, these cascades are modulated in relation to the cognitive workload required.

Seminal studies (31, 32) have explored EIB with fMRS, revealing how i) glutamate (26) is tied to more rapid changes (seconds to minutes) during cognitive tasks compared to GABA (minutes to tens of minutes); ii) and its temporal dynamics variations (33) depend on the complexity of the task. Consistent with this framing we found that EIB kinetics in high-order cognitive functions is characterized by a transient nature, increasing in the 2-Back condition. Parametrically manipulating WM proficiency (as seen by behavioral accuracy results of 1-back task ~98%) leads to a sudden rise in the excitatory activity of the prefrontal cortex (34–37), which is characterized by high glucose demand and oxidative metabolism (17, 38). Moreover, by looking separately at neurometabolites, we found a concomitant increase trend in Glx (35), thus pointing to a general up-regulation of the glutamatergic cycle during the attentive process or trends to decreased inhibition during learning/repeated stimulation (39–44). We observed inhibitory kinetics changes during the early periods of the 2-Back task (45, 46) as suggested by recent models of rapid shifts (16) supporting a biphasic GABAergic pattern (i.e., initial increase followed by a decline). This, along with glutamate fluctuations (47), suggests coordinated GABA-glutamate-glutamine cycling during WM engagement (45–50).

How do functional and neurochemical information streams contribute to our understanding of brain dynamics, and what are the temporal timescales of this relationship? We collect evidence that cognitive load affects the relationship between the temporal characteristics of FPN executive functioning and the kinetics of glutamate (Glx) and GABA+ balance, while expanding time-resolved insights into previously found associations [e.g., perceptual (26, 36) and highly demanding cognitive tasks (50)]. Greater temporal stability of the executive control network is thought to be beneficial in sustained attentive and WM processes (51, 52), particularly when characterized by dynamic reconfiguration and load-dependent modulation. We probe how temporal stable dynamics of prefrontal executive networks is associated with EIB kinetics and modulated across WM loads. Temporal features of FPN not only are parametrically stabilizing as a function of load condition (i.e., toughest cognitive performance) but are also highly aligned with EIB kinetics. The signature patterns of EIB kinetics and network stability of FPN probe a positive association in the highest cognitive load condition (e.g., 2-Back), whereas a reversed pattern is observed for the attentive-only task (e.g., 0-Back). Consequently, for high cognitive load conditions where FPN temporal properties are balanced, the EIB curve parametrically changes toward increased temporal gradient and reduced time-invariant evolution. As EIB kinetics increase over time, the excitation counterpart of the ratio (i.e., Glx) is presumably boosted relative to the inhibitory component, suggesting that metabolic characteristics of the DLPFC (19, 48) may be primarily related to glutamatergic neurons.

However, neurochemical-dependent changes in brain metabolite spectra are a subject of ongoing debate within the scientific community with various models offering different interpretations of these changes: (i) one perspective (17) suggests that these shifts are due to rapid exchanges between vesicular and cytosolic compartments, stabilizing within seconds; (ii) while another (18) posits that fluctuations are linked to slower metabolic processes related to energy metabolism rather than immediate neurotransmitter cycling. These differing viewpoints highlight the complexity of vascular responses and chemical signaling, which operate across vastly different time scales. While the effects on MR signals of the former are well characterized, a coherent description of neural signaling dynamics via MRS, and their interplay, remains to be fully established.

Our work adds to the field by closely looking at the timescale of these two key processes. The temporal stability of the FPN increases, as shown by the persistence probability curve, which rises after the third frame during the most cognitively demanding task, indicating that about 24 s of continuous WM activity is needed to detect this increment. Similarly, EIB changes are detectable as early as 12 s after baseline, reaching a steady state around 8 min into the observation period. These findings are consistent with previous studies that propose the coexistence of multiple timescales of the metabolic response: An initial rapid excitatory phase (lasting minutes (26)), followed by a slower phase (18, 32), where inhibition, on the order of tens of minutes, establishes a plateau. Moreover, the gradual emergence of the inhibitory component may be undetectable in static EIB analyses, as whole-acquisition averaging obscures the rapid Glx kinetics, which decay within seconds, while favoring the slower component persisting throughout the entire observation window. The temporal dynamics of the FPN during sustained WM load are tightly coupled to its network configuration. Other CAPs, although including large portions of the DLPFC, lack the persistence of the FPN, which correlate with EIB kinetics, suggesting a close relationship between EIB DLPFC regulation and WM networks. This mutual interaction seems to occur on similar timescales during the initial phase (with both curves showing the aforementioned rapid changes in the first frames, within 12 to 24 s from baseline), and remains elevated throughout the entire measurement window. The mechanism that supports both the elevated persistence on one side and EIB on the other may be due to a redefinition of the baseline equilibrium in EIB, driven by the cited progressive contribution of the inhibitory component, which limits the excitatory signaling likely stimulated by the energy demand required to support WM.

The convergence of temporal dynamics at both molecular and network levels naturally prompts the question: What underlying principle might account for such shared organization? Here, the recently proposed “common currency” framework (53–55) offers a particularly compelling interpretive lens. According to this view, temporality itself, expressed as shared timescales or dynamic motifs across molecular network, and cognitive domains, acts as a fundamental organizing principle of brain function (56). In our data, the alignment of EIB kinetics and FPN stable dynamics within similar temporal windows is unlikely to be coincidental; rather, it suggests a coordinated translation from neurochemical fluctuations to large-scale network reconfiguration, ultimately supporting cognitive operations such as WM. The “common currency” concept thus provides a bridge between the metabolic and systemic levels, and crucially, extends this link to the mental domain, allowing us to frame our findings as part of a broader temporal architecture that underpins cognition. In this sense, the temporal structure we observe is not simply a byproduct of measurement, but may represent the very scaffold through which the brain integrates molecular signals, network dynamics, and mental processes into coherent behavior.

Some limitations must be acknowledged. First, the target voxel in the DLPFC was proximate to the skull, potentially degrading spectra due to lipid artifacts and partial volume effects. Consequently (47–60), within the MRS sample, we excluded half of the initial sample size after quality checks, which may affect the accuracy and generalizability of our findings. We reported composite measures for Glx (Glu+Gln) due to the low reliability of linear combination modeling (29). A control voxel in an unrelated brain region was omitted due to time constraints; instead, we used a 0-Back control condition and a resting-state session to establish a subject-specific baseline for normalizing neuro-metabolite concentration changes across cognitive load conditions, reducing intersubject variability (61). Importantly, it should be explicitly stated that MRS measures total tissue concentrations of metabolites within the voxel, without compartmental specificity: meaning that it cannot distinguish between intracellular, extracellular, neuronal, or glial pools (62). This inherent limitation may obscure more nuanced neurochemical dynamics relevant to cognitive processes. Therefore, future studies should enhance spatial resolution of fMRS with advanced techniques like MRSI (63, 64), while also improving temporal measurement scales by incorporating proxy measures of EIB (e.g., Hurst exponent) from higher-resolution methods like EEG (13, 15, 16), acknowledging that only MRS provides in vivo noninvasive quantification of neurotransmitter concentrations, whereas functional and model-based surrogate (10) metrics offer valuable but indirect perspective.

In conclusion, using a peculiar combination of fMRS and fMRI, along with time-resolved analyses, we provide evidence of integrated temporal properties of executive networks and EIB kinetics in healthy volunteers. Understanding the temporal dimension through which metabolism and vascular response interact to drive *ad-hoc* reconfiguration of brain networks for information processing is crucial to possibly reframe cognitive disorders in terms of disrupted synchronicity or impaired coupling of neural signals (5), where present.

## Materials and Methods

### Participants.

A total of 36 healthy adult volunteers (17 females) participated in this study, with 24 (11 females) undergoing the full fMRI-fMRS experiment. The study received approval from the Ethical Committee of the University of Trento, and all participants provided written informed consent prior to participation in the study. Demographic data and potential confounding variables for the GABA-edited MRS (65) are presented in Table 2. Exclusion criteria included neurological or psychiatric disorders, current psychotropic medication use, high nicotine consumption (>1 cigarette/day), excessive alcohol intake (>14 units/week or >3 d/wk), and migraine with aura. Participants abstained from caffeine for 12 h before the scan. Due to the emerging nature of this research area, power analysis was based on prior studies (48) and performed using GPower (v3.1.9.7). The analysis indicated that a sample size of 22 was sufficient to detect a small to moderate within-subject main effect (f ≥ 0.20) across three tasks (*Experimental Design*) at the recommended power (0.80) and α = 0.05 for autocorrelated measures (r = 0.75). Therefore, an adequate number of participants were included in both the full sample and the fMRS subgroup.

**Table 2.** Demographic information of the healthy volunteer groups that underwent the parametric working memory task during functional MRI or during the combined functional MRI and MRS protocol (Fig. 4)

Sample	fMRI	fMRI-fMRS
Sample size	36	24
Age (years)	25.6 ± 3.2	25.0 ± 3.1
Gender	19 M, 17 F	13 M, 11 F
Education (years)	18.8 ± 2.5	18.8 ± 2.5
Handedness	5 left-handed	5 left-handed
Menstrual cycle	N.A.	Menstruations (1-5 d): 1 Proliferation phase (6-14 d): 3 Ovulatory phase (14-15 d): 1 Initial secretion (16-23 d): 2 Final secretion (14-28 d): 3 Amenorrhea: 1
Nicotine	N.A.	10 nicotine smokers

Details are reported using standard guidelines for MEGA-PRESS usage (65).

### Experimental Design.

**Fig. 4.**
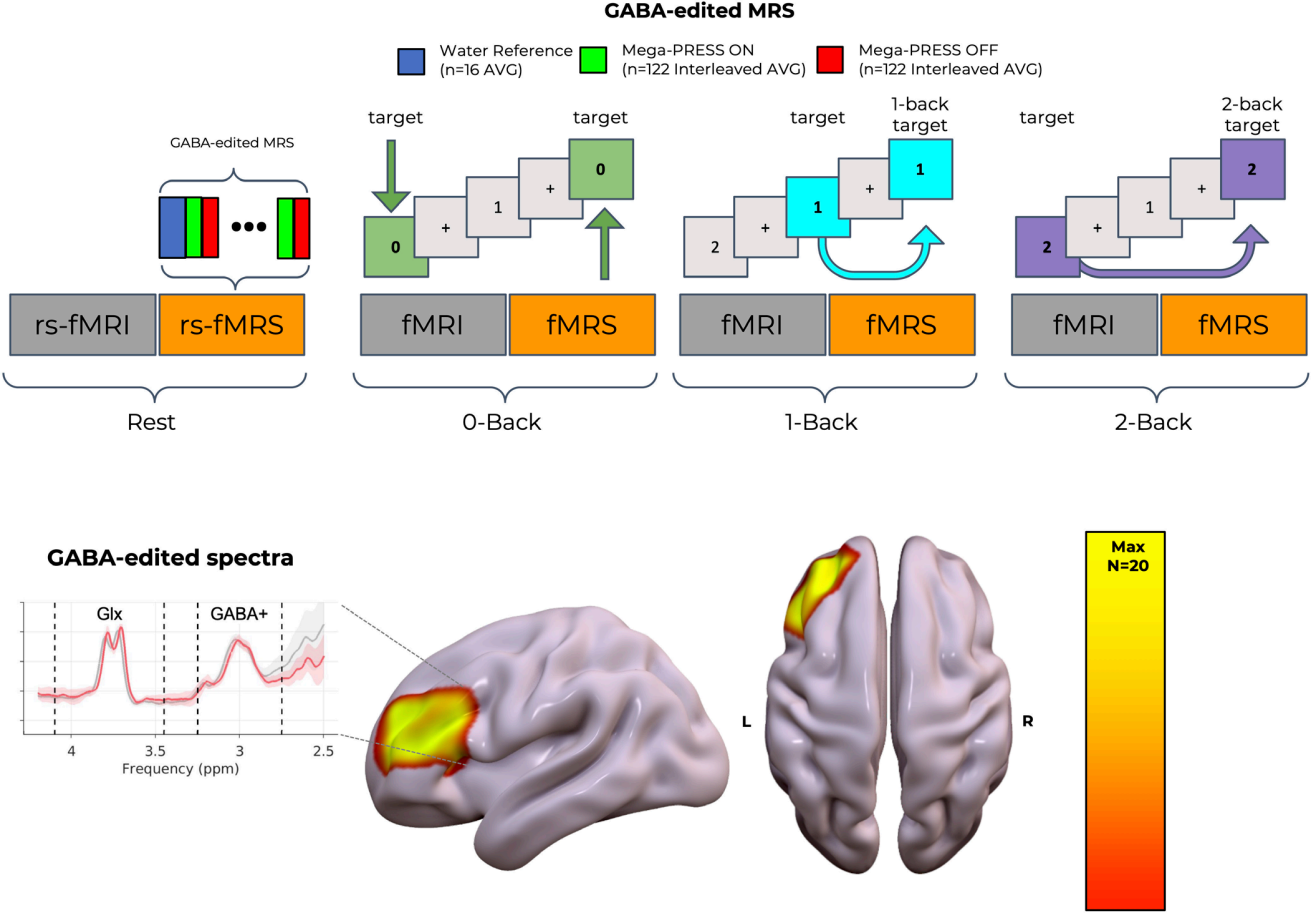
Exploring in vivo EIB kinetics as a function of cognitive load. The first row displays the experimental design consisting of four different sessions (session_1_ = Resting-state, session_2_ = 0-Back, session_3_ = 1-Back, and session_4_=2-Back), each one with interleaved fMRI-fMRS acquisitions lasting approximately 14 min in total per session. For each task session, visual stimuli were randomly selected as digits or letters. Before the beginning of each GABA-edited fMRS acquisition, a water reference sequence was acquired by disabling water suppression. The second row shows the probabilistic anatomical overlap of the MRS voxel placement across subjects in the left dorso-lateral prefrontal cortex (DLPFC) (Max: corresponds to 20 subjects overlapping with the total sample of 24 subjects) together with example mean spectra from the resting-state acquisition across all subject to identify GABA+ and Glx peaks. Abbreviations: MRS (magnetic resonance spectroscopy), AVG (averages), rs (resting state).

The experimental procedure aimed at noninvasive in vivo mapping of EIB from single-voxel MRS and brain function from BOLD fMRI while manipulating cognitive load in healthy volunteers Fig. 4). The design included four MRI sessions, each combining fMRI and fMRS acquisitions (66). The first session served as a baseline resting-state (TA: fMRI-fMRS ~ 14 min), where participants lay still, thought of nothing in particular, and fixated on a crosshair. The subsequent three sessions involved a visual WM task with increasing cognitive load: a 0-Back, 1-Back, and 2-Back task in the first, second, and third sessions, respectively. Task order remained consistent across subjects given previous studies about the timing of the expected metabolic response in the fMRS session (67) and to facilitate analyses of interindividual differences (68).

Each session utilized a block design with four blocks for both fMRI and fMRS acquisitions. To control for functional specialization in the WM system (69), stimuli consisted of letters and digits, remaining constant within each acquisition but varying between blocks. The assignment of stimulus type to acquisition type was pseudorandomized across sessions.

Regardless of task or stimulus type, each block contained 44 trials, each comprising a 2-s stimulus presentation followed by a 0.5-s crosshair display. In the 0-Back task, participants pressed a button upon seeing a target stimulus presented at the beginning of the block for 2 s. In the 1-Back and 2-Back tasks, they pressed a button when identifying stimuli that matched those presented one and two trials earlier, respectively. Each block included nine congruent trials where participants were expected to respond according to task instructions.

### Behavioral Analysis.

Behavioral data were collected during the three task-based sessions to assess the impact of cognitive load throughout the acquisition. Key metrics included reaction time (RT, in ms) for congruent stimuli, total accuracy (percentage of correct responses), hit rates (proportion of recognized congruent trials), and false alarms (proportion of incongruent trials identified as congruent) for each subject across sessions. Additionally, a sensitivity measure (70) (*d’*) was calculated based on the standardized difference between false alarm and hit rate distributions for each subject, with perfect scores adjusted using a log–linear approach (71).

To address trial-by-trial fluctuations in WM performance, we implemented a dynamic behavioral performance index that combines accuracy and RT for congruent trials, and response inhibition for incongruent trials. For congruent trials, high performance was defined as a correct and fast response [score approaching 1, with RT normalized between 400 to 800 ms based on the empirical RT distribution (72, 73)], while incorrect responses received a score of 0. For incongruent trials, successful inhibition (no response) was scored as 1, whereas false alarms were penalized by RT, resulting in scores ranging from approximately 0 to 0.25, with slower false alarms receiving lower scores. This scoring scheme generated a continuous performance metric (range 0 to 1) for each trial, enabling sensitive tracking of behavioral fluctuations over time (74). To align behavioral and neural analyses, the same sliding window parameters used for dynamic fMRS EIB estimation were applied to the behavioral data, and the area under the curve (AUC) was calculated for each session.

### MR Acquisition.

Data were acquired using a 3 T clinical MR scanner (MAGNETOM Prisma, Siemens Healthcare, Erlangen, Germany) with a 64-channel head-neck RF receive coil and parallel transmit RF (operating system VE11C). The optimized MR acquisition protocol included:

- 3D T1-weighted multiecho MPRAGE (75) (TR = 2.5 s, TEs = [1.69, 3.55, 5.41, 7.27 ms], TI = 1,100 ms, 1 mm isotropic voxels).
- Four sessions of BOLD 2D EPI (TR = 2 s, TE = 28 ms, 3 mm isotropic voxels, full brain coverage).
- Double-echo gradient echo sequence for distortion correction (TR = 682 ms, TE1/TE2 = 4.2/7.4 ms, 3 mm isotropic voxels).
- Four sessions of MEGA-PRESS MRS water reference (76–78) (TR/TE = 2000/68 ms, editing pulses = 1.9/7.5 ppm, bandwidth = 60 Hz, voxel size = 3 cm, spectral points = 2048, no VAPOR water suppression, 32 averages, TA = 2:08 min).
- Four sessions of MEGA-PRESS edited functional MRS (79) (TE/TR = 68/2,000 ms, automated shimming routine, editing pulses = 1.9/7.5 ppm, voxel size=3 cm isotropic, spectral points = 2048, VAPOR water suppression; resting-state = 192 averages, task = 224 averages; TA_REST_ = 6:50 min, TA_TASK_ = 7:40 min).

The MRS package was developed by Edward J. Auerbach and Małgorzata Marjańska and provided by the University of Minnesota under a C2P agreement. MRS data were collected using automated voxel placement (described in the *SI Appendix*, *Supplementary Materials and Methods*).

### Functional MRS Preprocessing.

MRS data were processed with Gannet v3.2.0 (https://github.com/markmikkelsen/Gannet.git) on Matlab version 9.5.0 (80). In order to process edited MRS, preprocessing steps included (57) i) gross head motion correction and ii) phase and frequency drift correction using a spectral registration algorithm (81). Following these corrections, the Fourier transform was computed to subtract frequency spectra between the ON and OFF spectra, which were then aligned and averaged (80). After preprocessing, neurometabolite spectra peaks were extracted by fitting for GABA+ and Glx (a compound of Glutamate and Glutamine) in the difference spectrum. The fitted spectra were corrected for the proportion of different tissue types in the voxel: gray matter (GM), white matter (WM), and cerebrospinal fluid (CSF). This involved coregistering the single voxel to the subject’s anatomical image with SPM12 and segmenting it into tissue types. Metabolite concentrations were quantified relative to an internal water reference, converting unitless signal intensity values into absolute concentrations with a weighting for tissue type percentages (62), allowing comparisons across subjects. Finally, the macromolecules (MM) contribution was considered by using the Gannet model (57), which assumes that 45% of the area of GABA+ peak is attributable to MM contribution (80).

### MRS Quality Control.

Data exclusion criteria were established for the processing methods (*SI Appendix*, *Supplementary Materials and Methods*), considering the fitting procedure type. Quality metrics included GABA signal-to-noise ratio (SNR) and fit error, as well as Glx SNR and fit error (60, 61). After computing fMRS quality metrics for each subject, a group distribution was obtained, and a z-score for each metric was calculated relative to this distribution. Data were excluded if the absolute z-score was greater than or equal to one in at least two quality metrics or in one session for the same metric. Data outliers were identified during the initial analysis described in the following paragraph and removed from further analysis (65).

### Neurometabolite Estimation: Static and Dynamic.

To evaluate changes in neurometabolite concentrations related to EIB under varying cognitive loads, a two-fold analysis was conducted. First, the overall average concentration was calculated for each full MRS acquisition session, separately for resting-state (192 averages: 96 ON and 96 OFF) and the 0-, 1-, and 2-Back conditions (224 averages: 112 ON and 112 OFF). These averages were processed together in Gannet using the GABAGlx model, with the mean concentration from the resting-state session serving as the subject-specific baseline for further analysis.

Second, to assess kinetics across sessions, as previously demonstrated of interest in the field (82–84), sliding window averages were computed. A window length of 60 averages (60 ON and 60 OFF) was used, ensuring adequate SNR for GABA+ estimates. This window was shifted in steps of 3 averages (12 s), resulting in 18 dynamic frames spanning cognitive conditions beside the resting-state, where they correspond to 12 frames. A small subset of averages was discarded from the resting condition to maintain consistent frame dimensions across sessions. To estimate individual fluctuations of EIB and associated metabolites over time, curves were normalized using the baseline mean value from the first frame of each cognitive condition, controlling for carryover effects and baseline shifts. Given the lower stability of the fitting due to the reduced number of averages, a median interpolation of outliers was applied.

To gain insights into temporal changes of neurometabolites, properties of EIB, Glx, and GABA+ across sessions were calculated. The limited number of time points made conventional kinetics properties (e.g., time to peak, slope, zero-crossing) sensitive to transients and local instabilities. Thus, a graph-theoretical approach was employed. The visibility graph method, described by Lacasa et al. (85), translates time series into graphs that retain the temporal properties of the original data. We considered 1) the average out-degree of the associated time-directed graph as a surrogate metric for the slope (lower average out-degree indicates time-dependent changes of EIB) and 2) the KLD—the distance between in- and out-degree distribution—as a proxy for stationarity of the EIB curve (86) (lower values are associated with time-invariant, or stationary, processes).

### Functional MRI Preprocessing.

The preprocessing of resting-state and n-back fMRI conditions was implemented using an FSL-based automated pipeline (https://github.com/tambalostefano/lnifmri_prep) that included common steps: 1) slice timing correction; 2) T1-weighted image tissue segmentation; 3) geometric distortion and head motion correction; 4) coregistration of the T1-weighted image to the time series. For the resting state fMRI sessions, further steps included: 5) regression from the time series of the 6 head motion parameters, white matter and CSF signals; 6) band-pass temporal filtering [0.01 to 0.10 Hz]. Both resting-state and task-based fMRI were warped into common space: 7) normalization to standard MNI template space; and 8) spatial smoothing 6 mm FWHM Gaussian kernel size.

### Functional CAP.

To estimate brain fMRI recurrent states associated with in vivo EIB and modulated by cognitive load levels, we utilized a coactivationpatterns (CAPs) framework for each fMRI session (https://github.com/MIPLabCH/TbCAPs). CAPs were derived by defining a seed in the left DLPFC (53) (10 mm radius) that matched the MNI coordinates of the voxel placement used in the fMRS scans, allowing for the identification of the seed BOLD time course. Timepoints where the seed BOLD activation exceeded a z-score of 1 were selected for further analysis (87). The fMRI frames from these timepoints were then processed using a k-means clustering procedure to reconstruct temporal assignments of the spatial patterns. To determine the optimal number of clusters (k), consensus clustering was performed (88). All frames assigned to the same state were averaged to reconstruct the corresponding CAP. Thus, absolute z-score CAP were extracted relative to the left DLPFC seed over limited periods of the time series, focusing on detecting recurring frontoparietal spatial patterns. Temporal metrics for each CAP (including the nonactive state, CAP_0_) were computed across different fMRI cognitive conditions by concatenating subjects from all sessions. These metrics included i) in-degree: likelihood of visiting a CAP from any other; ii) out-degree: likelihood of exiting a CAP toward another; iii) resilience: probability of remaining in the same configuration throughout the time series; iv) occurrences: frequency of a CAP’s reoccurrence over time; and v) Btw: importance of a CAP concerning the shortest paths between other CAP pairs (87). In this context, “stable dynamics” refers to the high occurrence and persistence of specific CAPs over the time course, indicating that certain patterns remain invariant and frequently recur throughout the observed period, rather than implying formal statistical stationarity of the underlying fMRI signal.

### Statistical Analysis.

Detailed description of the statistical methods exploited to produce the results is available in the *SI Appendix*, *Supplementary Materials and Methods*.

## Supplementary Material

Appendix 01 (PDF)

## Data Availability

Anonymized Scripts and processed anonymized data have been deposited in Zenodo (https://doi.org/10.5281/zenodo.15855362) (89).
